# Common Food Emulsifiers Impair Epithelial Barrier Integrity and Trigger Pro‐Inflammatory Responses and IgE Production

**DOI:** 10.1111/all.70473

**Published:** 2026-08-08

**Authors:** Duygu Yazici, Yuka Hayashi, Yagiz Pat, Jennifer Hotie, Mehmet Ali Balci, Sena Ardicli, Juan Felipe Lopez, Ozge Ardicli, Bingjie Zhao, Angel Maldonado, Can Zeyneloglu, Kenji Matsumoto, Manru Li, Beate Rückhert, Lihong Chang, Ismail Ogulur, Vahap Eldem, Raja Dhir, Mubeccel Akdis, Kari Nadeau, Oscar Palomares, Hideaki Morita, Cezmi A. Akdis

**Affiliations:** ^1^ Swiss Institute of Allergy and Asthma Research (SIAF) University of Zurich Davos Graubünden Switzerland; ^2^ Department of Allergy and Clinical Immunology National Research Institute for Child Health and Development Tokyo Japan; ^3^ Department of Biology, Faculty of Science Istanbul University Istanbul Turkiye; ^4^ Department of Genetics, Faculty of Veterinary Medicine Bursa Uludag University Bursa Turkiye; ^5^ Division of Food Processing, Milk and Dairy Products Technology Program, Karacabey Vocational School Bursa Uludag University Bursa Turkiye; ^6^ Department of Biochemistry and Molecular Biology, School of Chemistry Complutense University of Madrid Madrid Spain; ^7^ Department of Environmental Health Harvard T.H. Chan School of Public Health Boston Massachusetts USA; ^8^ Allergy Center National Center for Child Health and Development Tokyo Japan

**Keywords:** DATEM, emulsifiers, epithelial barrier, gastrointestinal tract, gut‐on‐a‐chip, soy lecithin

## Abstract

The global rise in allergic and autoimmune diseases since the 1960s has been associated with environmental disruptions of epithelial barriers, particularly in the gastrointestinal tract. Among the implicated factors are food emulsifiers, which have become increasingly prevalent in processed foods such as peanut butter. This study investigates the biological effects of two commonly used food emulsifiers in peanut butter, soy lecithin and diacetyl tartaric acid esters of mono‐ and diglycerides (DATEM), on gastrointestinal tissue responses and immune responses using advanced in vitro gut‐on‐a‐chip models, human adult stem cell‐derived colon organoids‐on‐a‐chip, and murine models. Both emulsifiers induced dose‐dependent cytotoxicity and disrupted epithelial barriers, as demonstrated by reduced transepithelial electrical resistance and disorganization of zona occludens‐1 at daily‐exposure doses. Transcriptomic and proteomic analyses revealed activation of pro‐inflammatory pathways including TNF, NF‐κB, the unfolded protein response, and cell death by apoptosis. Soy lecithin additionally induced oxidative stress. Systemic responses were confirmed by increased pro‐inflammatory cytokines in serum and immune activation in peripheral blood mononuclear cells (PBMCs). Notably, both emulsifiers stimulated serum total IgG, soy lecithin induced IgE in mice upon feeding, and DATEM enhanced IL‐4‐induced IgE production in human PBMCs, suggesting a role in allergy development. These findings challenge the presumed safety of soy lecithin and DATEM and highlight their potential contribution to immune dysregulation and epithelial barrier dysfunction, which may initiate or exacerbate food allergy and gastrointestinal inflammatory diseases.

## Graphical abstract

- Soy lecithin and DATEM, the two commonly used food emulsifiers introduced into many foods including peanut butter and puffed bread, in the 2000s, impair intestinal epithelial barrier integrity in human gut‐on‐a‐chip and induced pluripotent stem cell colon organoid models.
- Both emulsifiers activate NF‐κB/TNF‐driven inflammatory pathways and induce stress responses and apoptosis in epithelial cells, with soy lecithin additionally triggering oxidative stress and suppressing aryl hydrocarbon signaling in gut epithelial cells.
- Oral exposure to emulsifiers within the range of daily doses induces local gastrointestinal and systemic inflammatory responses in mice and increases IgE and IgG responses, suggesting a role in the development of allergies. AhR, aryl hydrocarbon receptor; CCL, chemokine C‐C motif ligand; CTLA4, cytotoxic T‐lymphocyte associated protein 4; Ctrl, control; CXCL2, C‐X‐C motif chemokine ligand; DATEM, diacetyl tartaric acid ester of mono‐ and diglycerides; FOXO1, forkhead box protein O1; Ig, immunoglobulin; NF‐κB, nuclear factor kappa B; PPP1R2, phosphatase inhibitor 2; PRDX5, peroxiredoxin‐5; RNAseq, RNA sequencing; SNAP29, synaptosomal‐associated protein 29; TER, transepithelial electrical resistance; TGF, transforming growth factor; TNF, tumor necrosis factor; TNFRSF11B, osteoprotegerin.

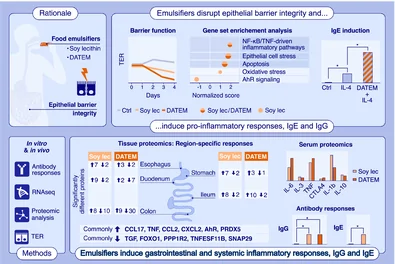

## 1 Introduction

Since the 1960s, the incidence of allergic diseases has risen dramatically and reached epidemic levels [1]. While the incidence has plateaued in industrialized countries [2, 3], it is still on the rise in developing regions, which are undergoing westernization, socioeconomic affluence, and urbanization [4]. A significant increase in autoimmune and some neuropsychiatric diseases also started in the 1960s and is on the rise in developing countries, coinciding with the allergy epidemic [5, 6, 7, 8, 9, 10]. The epidemic started with the substantially rising prevalence of food allergies (FAs), eosinophilic esophagitis, and drug‐induced anaphylaxis after the 2000s [11, 12, 13]. The pathogenesis of these diseases has been associated with defective epithelial barriers in the affected organs or in the gut [14, 15]. The epithelial barrier has been shown to be impaired by a variety of factors that have significantly increased in our environment since the 1960s. These include ground‐level ozone, diesel exhaust, particulate matter, cigarette smoke, laundry and dishwasher detergents, household cleaners, surfactants, enzymes, and emulsifiers in processed foods [16, 17, 18, 19, 20, 21, 22, 23, 24, 25, 26, 27, 28, 29].

Over the past 20 years, the prevalence of peanut allergy (PA) has substantially increased, reaching 1.4%–2% in both Europe and the US [30]. In the meantime, the point prevalence of Food allergies (FAs) in general has increased from very low levels to 13% of westernized populations [31, 32]. PA is linked to potentially fatal anaphylaxis. Every year, between 7% and 14% of PA patients unintentionally come into contact with peanuts, and between one‐third and one‐half of them are likely to develop anaphylaxis [33]. A study of food‐induced anaphylaxis‐related pediatric emergency department visits and hospital admissions from 2008 to 2012 revealed a substantial rise in these cases, with an average annual increase of 30% reported for incidents caused by peanuts [34]. It is uncertain what is causing PA and anaphylaxis to become so common, particularly in the last two decades, given that they still have very low rates in China [35], Turkiye [36] and Israel [37] although individuals in these regions are exposed to substantial amounts of native peanuts. Several theories encompassing both genetic and environmental variables have evolved to explain this recent development. According to the hygiene hypothesis, a rise in allergies is being caused by a reduction in early‐life exposure to certain infectious agents and microbial products [38]. Another idea focuses on the timing of introducing peanuts to newborns or children, as well as the consumption of peanuts by mothers during pregnancy and nursing. Increased peanut intake, the usage of non‐food products containing peanuts, and the early introduction of proteins like soy that may react with one another could all be contributing reasons. Third, a major change that took place in common peanut butter processing methods, such as roasting, and particularly emulsifying, and mixing additives with peanuts, may increase their antigenicity and contribute to the rising prevalence of peanut allergy [39].

Emulsifiers first began to be added to peanut butter in the 1970s, a practice that became more and more common afterwards [40]. Since the 2000s, the inclusion of emulsifiers (Table S1) in peanut butter became the norm [41], which coincides with the start and increase of PA and anaphylaxis. Peanut butter is made from peanut paste and has a creamy texture [42]. Emulsifiers are added at the grinding stage of production during “no‐stir” peanut butter manufacturing to prevent oil separation and particle settling [43]. After all the components have been evenly distributed, the peanut butter's texture is improved by cooling, which causes fat crystallization, creating a network of fat crystals that immobilizes free oil and delays phase separation [43]. The most widely used emulsifiers in peanut butters and related products are lecithin, palm oil, and esters of mono‐ and diglycerides [44]. In this study, we investigated soy lecithin and diacetyl tartaric acid esters of mono‐ and diglycerides (DATEM), as these were one of the first emulsifiers to be introduced into peanut butter and are still commonly used in many countries (Table S1). Based primarily on research in mice, it has been shown that high doses or chronic consumption of food emulsifiers can cause negative health effects by causing gut inflammation, microbial dysbiosis, obesity, and metabolic dysregulation [14, 45]. As emulsifiers are known to adversely affect epithelial cells, it is important to investigate the underlying mechanisms of intestinal epithelial barrier defects in response to other commonly used emulsifiers. Therefore, in this study, we examine the biological effects of two widely used peanut butter emulsifiers, soy lecithin and DATEM, on intestinal epithelial integrity and immune responses, utilizing advanced in vitro gut‐on‐a‐chip models, human adult stem cell‐derived colon organoid‐on‐a‐chip models, and in vivo murine models.

## 2 Materials and Methods

### 2.1 Analyses of Cell Viability

Quality control and endotoxin detection of all reagents were performed. The PyroGene Recombinant Factor C Endpoint Fluorescent Assay (Lonza Bioscience) was used to assess the endotoxin levels in the emulsifiers according to the manufacturer's protocol. Serial dilutions of soy lecithin (cat no. 1016–50, Modernist Pastry, USA) and DATEM (cat no. 100085–39‐0, Henan Chemsino Industry, China) were prepared and the fluorescence was measured at the beginning and end of the 1‐h incubation using a fluorescent microplate reader (Berthold Technologies, Germany) with excitation/emission wavelengths of 380/440 nm. To validate the assay's performance, each dilution was spiked with a known concentration of endotoxin standard, and the recovery of the spike was calculated. Recovery values between 50% and 200% were considered acceptable, indicating the assay's functionality in detecting endotoxin. Endotoxins were not found at detectable levels (< 0.01 EU/mL).

Caco‐2 cells were cultivated in 96‐well plates at a density of 25,000 cells/well and incubated overnight at 37°C in a humidified incubator with 5% CO_2_. Emulsifiers were applied to the cultures for 24 h. After removing the medium, PBS was used to wash the cells. MTT solution (Invitrogen) was added to new medium and incubated at 37°C for 4 h in a CO_2_ incubator. After incubation, MTT solution was removed from the wells and 100 μL of DMSO was added to dissolve the formazan crystals. The plate was shaken vigorously for 10 min, and the absorbance was measured at 520 nm using a microplate reader (Berthold Technologies, Germany).

### 2.2 Epithelial Barrier Integrity Assessment

A gut‐on‐a‐chip system was used to assess the effect of emulsifiers on colon epithelial cells [28, 29]. Caco‐2 cells were purchased from Sigma–Aldrich (#86010202) and were used between passages 20 and 45. Cells were cultured as a monolayer in Eagle's minimal essential medium (EMEM, ATCC) supplemented with 10% fetal calf serum (FCS, Sigma‐Aldrich), 1% penicillin/streptomycin (Sigma‐Aldrich), 1% non‐essential amino acids (Sigma‐Aldrich), and 1% sodium pyruvate (Sigma‐Aldrich) in a 150‐cm^2^ T‐flask in a humidified incubator at 37°C in 5% CO_2_. Gut‐on‐a‐chip cultures were established with OrganoReady plates (Mimetas BV, Leiden, The Netherlands) following the manufacturer's protocol. On day 8 of gut‐on‐a‐chip cultures, the chips were exposed to various concentrations of emulsifiers dissolved in culture medium for 4 days. Transepithelial electrical resistance (TER) was measured using an OrganoTEER device (Mimetas) once daily for 4 days.

Adult stem cell‐derived colon organoid‐on‐a‐chip models (OrganoReady colon organoid plates, Mimetas, NL) were used to investigate the effects of emulsifiers more in vivo relevantly. TER was measured once daily for 3 days.

### 2.3 Immunofluorescence Staining of Tight Junctions

The localization of tight junction (TJ) proteins in colon organoid‐on‐a‐chip cultures was assessed by immunofluorescent antibody labeling. The cells were fixed with 4% paraformaldehyde (Fluka, St. Louis, Mo, USA) for 10 min at room temperature and then permeabilized with 0.1% Triton X‐100 (Acros Organics, Geel, Belgium) and 0.02% SDS (Sigma‐Aldrich) in PBS for 5 min. The cells were incubated in a blocking buffer containing 10% goat serum (Dako Cytomation, Glostrup, Denmark) in 1% BSA/PBS for 15 min, followed by incubation with a mouse anti‐occludin (Invitrogen) and/or a rabbit anti‐claudin‐1 (Invitrogen). The cells were then incubated with secondary antibodies for 30 min at room temperature. DAPI was added together with the secondary antibodies. Immunolocalization and the integrity of TJ proteins were visualized, and images were obtained using a Zeiss LSM 780 (Carl Zeiss Microscopy GmbH, Oberkochen, Germany).

### 2.4 NF‐κB Reporter Assay

NF‐κB reporter (Luc)‐HCT‐116 cells were cultured in McCoy's medium (Thermo Fisher Scientific) supplemented with fetal calf serum and penicillin–streptomycin. A total of 5000 of these cells were seeded into a 96‐well white plate and treated with emulsifiers at 37°C in a humidified incubator with 5% CO_2_ for 24 h. The ONE‐Step Luciferase Assay System (BPS Bioscience) was used according to the manufacturer's recommendations to detect NF‐κB stimulation. A luciferase assay working solution in an amount equal to the volume of the cell culture medium was added to the 96‐well plates after 24 h of treatment. The plate was gently rocked for 20 min, and the firefly luminescence was measured with a microplate reader.

### 2.5 Aryl Hydrocarbon Receptor (AhR) Reporter Assay

AhR reporter human HepG2 cells were grown and maintained according to the manufacturer's instructions in EMEM, 10% FCS, 1 × non‐essential amino acids medium, and 100 μg/mL Zeocin (InvivoGen). A total of 20.000 of these cells were seeded into a 96‐well plate and treated with emulsifiers at 37°C in a humidified incubator with 5% CO_2_ for 24 h without Zeocin. The QUANTI‐Luc 4 Lucia/Gaussia detection system (Invivogen) was used according to the manufacturer's recommendations to detect AhR activation. Twenty microliters (20 μL) of the supernatant from stimulated HepG2‐Lucia AhR cells was transferred to a white (opaque) 96‐well plate, and 50 μL of QUANTI‐Luc 4 Reagent working solution was added. The Luminescence was measured immediately with a microplate reader.

### 2.6 Reactive Oxygen Species (ROS) Determination

5‐(and‐6)‐chloromethyl‐2′,7′‐dichlorodihydrofluorescein diacetate, acetyl ester (CM‐H_2_DCFDA) (Invitrogen) was used for the detection of ROS generation within cells following emulsifiers treatment. Caco‐2 cells were seeded in 96‐well plates at a density of 10.000 cells/well concentration and incubated overnight at 37°C humidified incubator with 5% CO_2_. The cells were treated with 20 μM CM‐H_2_DCFDA for 45 min. After CM‐H_2_DCFDA treatment, the cells were washed and treated with different doses of emulsifiers for 24 h in PBS supplemented with 10% FCS. Absorbance was measured at an excitation wavelength of 485 nm and an emission wavelength of 535 nm with a microplate reader.

### 2.7 Mouse Models of Peanut Butter Exposure

BALB/c mice were purchased from Sankyo Labo Service Corporation Inc. All mice were housed under specific‐pathogen‐free conditions in an environmentally controlled animal room at the National Research Institute for Child Health and Development. All animal experiments were approved and performed in accordance with the Guidelines of the Animal Care and Use Committee of the National Research Institute for Child Health and Development, Tokyo, Japan (A2012‐004‐C13).

Six‐week‐old female BALB/c mice were fed peanut butter with or without emulsifiers once per week for six weeks (Table S3). Eight mice were used for each condition (Table S3). Mice were randomly assigned to experimental groups prior to the start of the experiments. All outcome assessments were performed by an investigator, who was blinded to group allocation. The proper amount of peanut butter powder shown in Table S2 was weighed (Powdered Peanut Butter, Bell Plantation) and mixed with the respective mL of ultrapure water (Milli‐Q Advanced Water Purification System, MilliporeSigma). The required amounts of emulsifiers were weighed as well for each dose group and mixed with the peanut butter. The prepared mixture was stored at 4°C for no longer than 2 weeks. Three grams (3 g) of peanut butter were presented to individual mice in cell culture dishes for self‐administration (Table S3). The animals were sacrificed 2 days after the last exposure. Serum samples were collected from each mouse and stored at −30°C. Colon, ileum, jejunum, stomach, and esophagus tissue samples were put in RNAlater (RNAlater Stabilization Solution, Thermo Fisher Scientific) and stored at −80°C for further analysis.

### 2.8 RNA Isolation and Sequencing

RNA from the gut‐on‐a‐chip cultures was harvested at 24 h of emulsifier exposure. After feeding the mice with emulsifiers for 6 weeks, the esophagus and colon tissues were collected in RNAlater and stored at −80°C. Total RNA was extracted and purified with the RNeasy Plus Micro Kit (Qiagen, Hilden, Germany) according to the manufacturer's protocol. A NanoDrop 2000 spectrophotometer (Thermo Fisher Scientific) and 2200 TapeStation Automated Electrophoresis System (Agilent Technologies, Santa Clara, CA, USA) were used to determine the quantity and quality of the isolated RNA.

Samples with RNA integrity numbers of 9.4 or more were chosen for sequencing. RNA sequencing (RNA‐seq) was performed on an Illumina NovaSeq 6000 with the TruSeq RNA Stranded Sample Prep Kit (Illumina, San Diego, CA, USA). Raw sequencing reads were first subjected to quality control checks and trimming using BBDuk, which removes adapter sequences and low‐quality bases. The high‐quality, trimmed reads were then aligned to the human reference genome (GRCh38.p14) using the Subread aligner, ensuring accurate mapping of the reads. Gene‐level expression was quantified using featureCounts, part of the Rsubread package. This method counts reads mapping to known gene features annotated in Gencode Release 46, providing robust expression metrics.

### 2.9 Targeted Proteomics Analysis

Gut‐on‐a‐chip, human ASC‐derived colon organoid‐on‐a‐chip, mouse serum, mouse tissue samples, and peripheral blood mononuclear cells (PBMCs) were used for targeted proteomics analysis. Culture medium from organ‐on‐a‐chips treated with emulsifiers for 48 h was used. The samples were analyzed using the proximity extension assay (Olink) targeted proteomics technology (Uppsala, Sweden). For the gut‐on‐a‐chip model, inflammation, immune response, organ damage and metabolism panels were used (366 proteins). For the human ASC‐derived colon‐organoid‐on‐a‐chip model, inflammation and immune response panels were used (182 proteins). For mouse serum, the Target Mouse Cytokine panel (48 proteins) was used, and for mouse tissue samples, both the Target Mouse Cytokine panel (48 proteins) and the Mouse Exploratory panel (92 proteins) were used.

### 2.10 IgG and IgE Detection in PBMCs

To measure serum total IgG and IgE levels of mice fed with emulsifiers, Mouse Total IgG ELISA (Thermo Fisher Scientific) and Mouse Total IgE ELISA (Thermo Fisher Scientific) kits were used according to the manufacturer's protocol.

For the detection of IgE mRNA in PBMC samples, blood samples from healthy individuals were collected in heparinized tubes; subsequently, they were diluted in PBS and centrifuged with Ficoll Histopaque (Sigma–Aldrich, St. Louis, MO, USA) to visualize and extract the mononuclear cell layer. All participant materials were collected under the ethical permissions of the local institutional ethics committees, and the study was conducted following the Declaration of Helsinki for ethics approval and consent. PBMCs were washed three times with PBS/EDTA and resuspended in 1 mL of RPMI 1640 (Lonza, Basel, Switzerland) medium supplemented with 10% heat‐inactivated FCS (Sigma‐Aldrich), 1 × penicillin/streptomycin (Sigma–Aldrich, St. Louis, MO, USA), 1 × vitamins (Sigma–Aldrich, St. Louis, MO, USA), 1 × sodium pyruvate solution (Sigma–Aldrich, St. Louis, MO, USA), and 1% MEM non‐essential amino acid (NEAA) (Sigma–Aldrich, St. Louis, MO, USA) solution. Cells were counted with LUNA‐II Automated Cell Counter (Logos Biosystems, Gyeonggi‐do, South Korea) and seeded into 24‐well plates as 1 × 10^6^ cells per well in complete RPMI 1640. PBMCs treated with emulsifiers for 5 days were used to check the IgE mRNA levels. IL‐4 (PeproTech, 25 ng/mL) was used to induce IgE production. After 5 days, the cells were collected, and 350 μL of buffer RLT with 1% beta‐mercaptoethanol was added. The mixture was vortexed and stored at −80°C. Total RNAs were purified with the RNeasy Plus Micro Kit (Qiagen, Hilden, Germany) according to the manufacturer's protocol. Total RNA was converted to complementary DNA with the RevertAid RT Kit (Thermo Fisher Scientific, Waltham, MA, USA) and random hexamers according to the manufacturer's recommendations. Gene expression (5 ng cDNA per well) was assessed by qRT‐PCR using 5 μM of appropriate primers (IgE epsilon I forward primer, 5′‐ACA CAT CCA CAG GCA CCA AA‐3′: IgE IgHCE reverse primer, 5′‐TTG CAG CAG CGG GTC AA‐3′) and SYBR Green/ROX qPCR Master Mix (Thermo Fisher Scientific, Waltham, MA, USA), performed on a QuantStudio 7 Flex Real‐Time PCR System (Thermo Fisher Scientific, Waltham, MA, USA). Gene expression was normalized to elongation factor 1α (EEF1A1) and presented as relative quantification calculated using ΔΔCt method, using the untreated cells as the calibrator.

### 2.11 Data and Statistical Analyses

All statistical analyses and associated figures were generated using GraphPad Prism software (version 9.0) or the R package. Differences among groups were analyzed using the Kruskal–Wallis test followed by Dunn's post hoc test and a *p*‐value < 0.05 was considered statistically significant.

Proximity extension assay data were analyzed using R (version 4.2.0). Differential expression analysis of proteins was achieved using the OlinkAnalyze package (version 3.8.2). Benjamini–Hochberg false discovery rate corrected *q*‐values were calculated to correct for multiple testing in all parts of the differential expression analysis. Proteins with an adjusted *p*‐value < 0.05 were considered significantly differential biomarkers.

## 3 Results

### 3.1 Emulsifiers Cause Gut Epithelial Barrier Damage and Cell Death

The cytotoxicity of both soy lecithin and DATEM on gastrointestinal epithelial cells was assessed at levels consistent with the reported usage (Table S4), which ranged between 10 and 50 mg/kg for lecithin and 5 and 56 mg/kg for DATEM in nut spreads, cocoa and chocolate products, confectionery, bread and rolls, processed meats, food supplements, and chewing gum. For the daily usage of both emulsifiers, it is quantum satis (the amount that is sufficient), meaning that there is no maximum permitted dose [46, 47]. We analyzed their toxicity in Caco‐2 cells and investigated their functional effects on epithelial barrier integrity by measuring TER in the gut‐on‐a‐chip model. The results demonstrated that soy lecithin showed cytotoxicity at 25 mg/mL (Figure 1A) and caused significant epithelial barrier damage at 4‐ to 16‐times lower concentrations in a dose‐dependent manner (Figure 1B). DATEM exhibited cytotoxicity at 4 mg/mL (Figure 1A) and decreased the barrier integrity at 4‐ to 16‐times lower concentrations in a dose‐dependent manner (Figure 1B). To further confirm the detrimental effects of the emulsifiers, we used a human ASC‐derived colon organoid‐on‐a‐chip model. In line with the findings from the gut‐on‐a‐chip model, both soy lecithin and DATEM disrupted the epithelial barrier in a dose‐dependent manner (Figure 1C).

**FIGURE 1:**
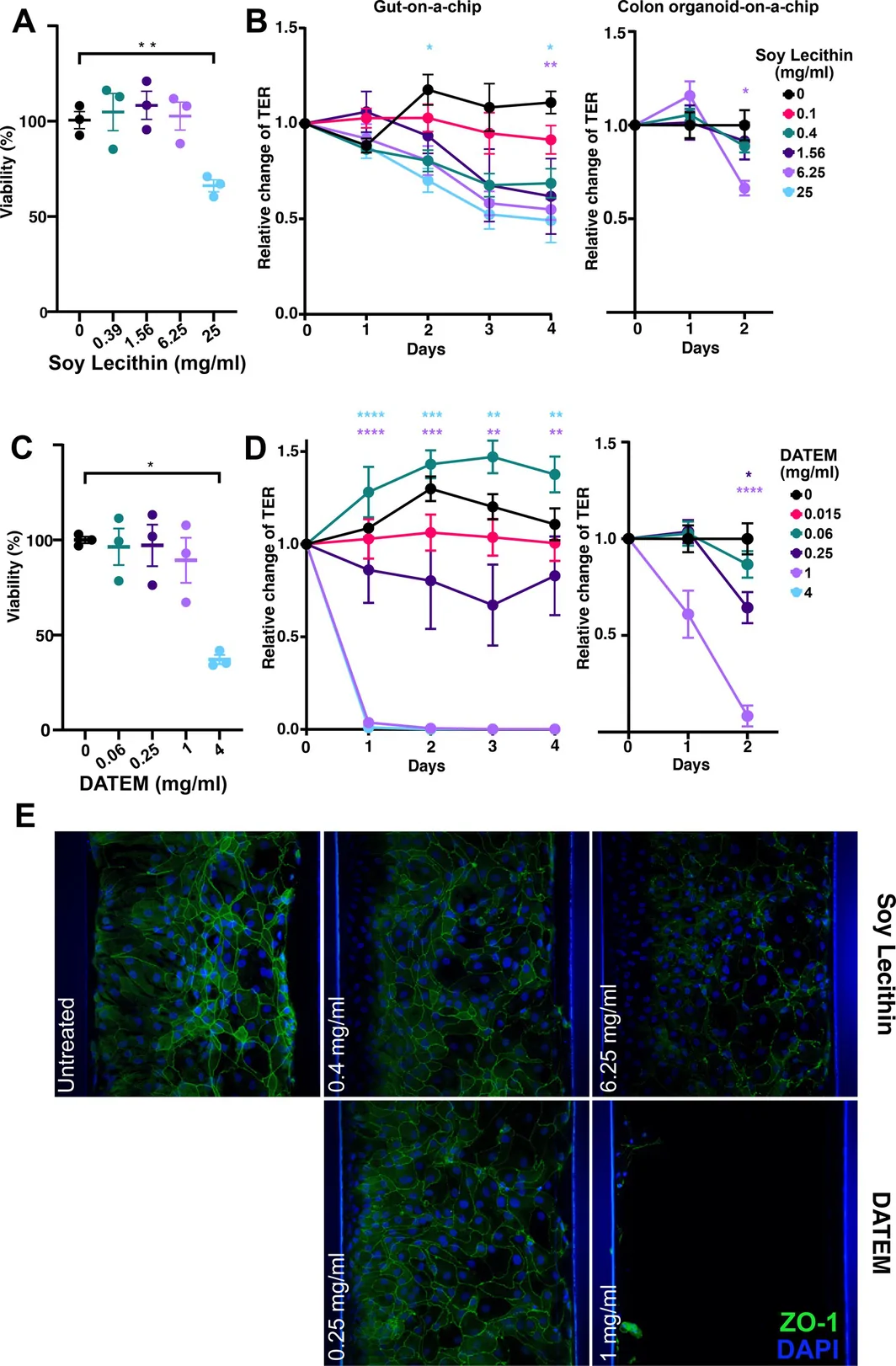
Soy lecithin and DATEM induce cytotoxicity and decrease barrier integrity. (A) Viability after a 1 day of treatment with different concentrations of soy lecithin and DATEM was assessed using the MTT assay in monolayer cultures of Caco‐2 cells. (B) Transepithelial electrical resistance (TER) was measured every 24 h for 4 days in the gut‐on‐a‐chip model and (C) for 2 days in the colon organoid‐on‐a‐chip model after exposure to soy lecithin and DATEM. (D) Representative immunofluorescence images of the colon organoid‐on‐a‐chip exposed to 0.6 mg/mL and 6.25 mg/mL soy lecithin and 0.25 mg/mL and 1 mg/mL DATEM for two days. Green represents ZO‐1 staining, while blue shows nuclear staining. Data are presented as the mean ± SEM from three independent experiments conducted at different times. *p < 0.05, **p < 0.01, ***p < 0.001, ****p < 0.0001, Kruskal–Wallis test with Dunn's post hoc test.

To extend our studies on epithelial barriers, immunofluorescence staining of the tight junction‐associated protein, ZO‐1, was performed in the gut‐on‐a‐chip model. After exposure to both soy lecithin and DATEM, dose‐dependent changes in the expression levels, integrity, and organization of ZO‐1 were observed (Figure 1D). Even after low‐dose treatment with soy lecithin (0.4 mg/mL) and DATEM (0.25 mg/mL), an irregular or reduced staining of ZO‐1 was detected. In untreated controls, ZO‐1 showed continuous staining and well‐defined localization around cell borders, indicating the presence of intact tight junctions (Figure 1D). These stainings demonstrate the impairment of epithelial barrier integrity with soy lecithin and DATEM, consistent with the TER results.

### 3.2 Soy Lecithin Induces Oxidative Stress, Inflammation and Cell Death as Observed in Transcriptome and Proteome of Gut‐On‐a‐Chip

In‐depth analyses of the transcriptomic and proteomic effects of both emulsifiers were investigated in gut‐on‐a‐chip and mouse models. First, dose‐dependent transcriptomic analyses were performed to assess the effects of soy lecithin on the gut‐on‐a‐chip model after 24 h exposure. Principal component analysis (PCA) reduced the dataset to two dimensions, with PC1 and PC2 explaining 49.2% and 11.5% of the total variance, respectively. The PCA plot showed clear clustering according to the groups, indicating distinct differences between the two doses and exposed and unexposed conditions (Figure 2A). A heatmap of the top 500 variable genes showed the dose‐dependent response (Figure S1A). Exposure to 6.25 mg/mL of soy lecithin resulted in differential expression (*p* < 0.05) of 3501 genes, as seen in the volcano plot (Figure S1B). Gene set enrichment analysis (GSEA) of 6.25 mg/mL soy lecithin exposure revealed significant upregulation of TNF‐α signaling, the response to unfolded protein, apoptosis, response to cytokines and chemokines, the complement pathway, mTOR signaling, epithelial–mesenchymal transition, and wound healing, and their sub‐heatmaps have demonstrated the major genes involved (Figure 2B,C).

**FIGURE 2:**
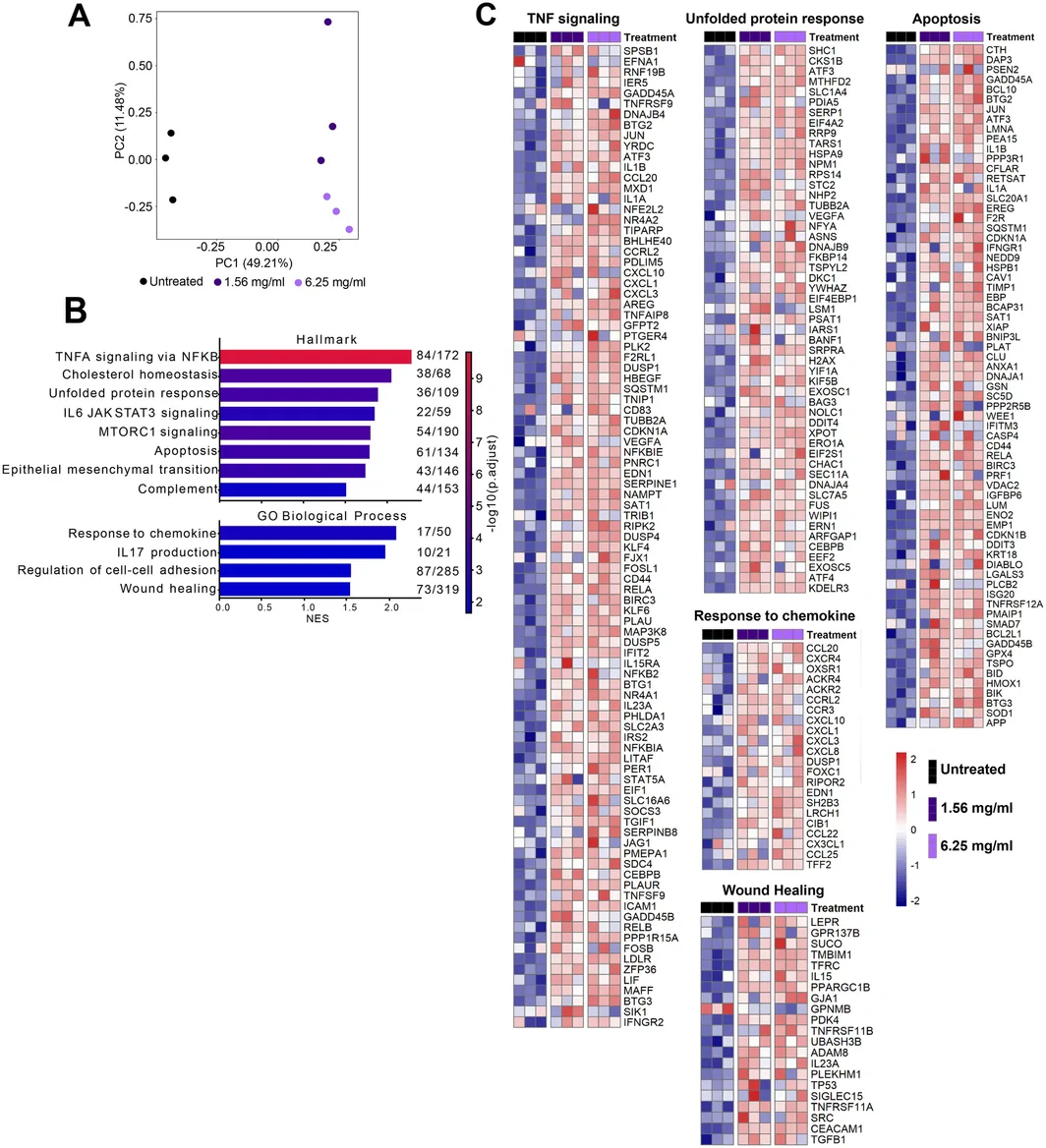
Transcriptome analysis of gut epithelial cells in response to soy lecithin in a gut‐on‐a‐chip model. (A) Principal component analysis (PCA) shows three distinct groups. The black dots represent control samples, while dark and light purple show 1.56 mg/mL and 6.25 mg/mL, respectively. (B) GSEA of 6.25 mg/mL soy lecithin exposure. Hallmark and Gene Ontology Biological Process (GOBP) databases were used. TNF‐α signaling via Nf‐κB (M5890), cholesterol homeostasis (M5892), unfolded protein response (M5922), IL‐6/JAK/STAT3 signaling (M5897), mTORC1 signaling (M5924), apoptosis (M5902), epithelial–mesenchymal transition (M5930), complement (M5921), response to chemokine (GO:1990868), IL‐17 production (GO:0032620), regulation of cell–cell adhesion (GO:0022407), and wound healing (GO:0042060). (C) Sub‐heatmaps of the pathways and their respective genes after 24 h of treatment with soy lecithin.

To check the proteome differences upon exposure to soy lecithin in the gut‐on‐a‐chip model, targeted proteomic analyses were performed with four different panels, namely, immune response, inflammation, metabolism, and organ damage, by using a proximity extension assay (Figure 3A). Consistent with the transcriptomic results, targeted proteomic analysis showed increased secretion of TNF‐α signaling‐related mediators such as IL8, CXCL1, JUN, IRAK1, CASP‐8, AXIN1, AREG, and LIF (Figure 3A). To support and complement these data, we conducted targeted proteomics with a human ASC‐derived colon organoid‐on‐a‐chip model and observed an increase in important cytokines, chemokines, and alarmins such as TNF, AREG, IL17C, CCL20, CCL4, and IL33 on treatment with soy lecithin (Figure S1C).

**FIGURE 3:**
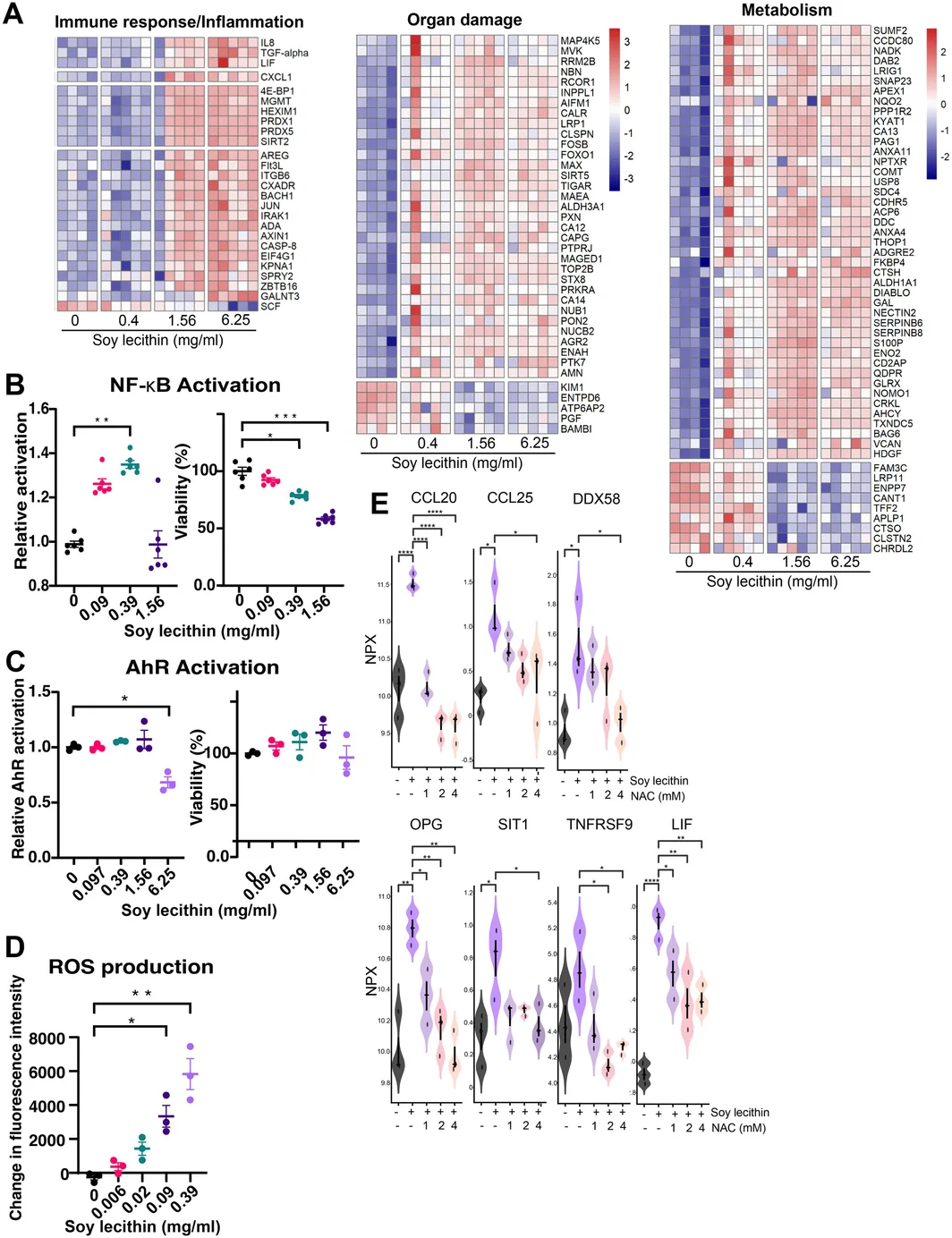
Soy lecithin induces NF‐κB activation and inflammation through oxidative stress. (A) Heatmap of targeted proteomic analysis of the gut‐on‐a‐chip model treated with soy lecithin. Immune response, inflammation, metabolism, and organ damage panels were used. (B) An NF‐κB activation assay was performed with the HCT‐116 reporter cell line. Cells were treated with soy lecithin for 24 h. Data are shown as relative NF‐κB stimulation. (C) An AhR activation assay was performed with the HEK reporter cell line. Cells were treated with soy lecithin for 24 h. Data are shown as relative AhR stimulation. (D) Reactive oxygen species (ROS) release was performed with Caco‐2 cells. Cells were treated with soy lecithin for 6 h. Data are shown as changes in fluorescence intensity. (E) Violin plots showing the targeted proteomic analysis of the gut‐on‐a‐chip model treated with soy lecithin and NAC. Data are presented as the mean ± SEM from three independent experiments conducted at different times. *p < 0.05, **p < 0.01, ***p < 0.001, ****p < 0.0001, Kruskal–Wallis test with Dunn's post hoc test.

To support the findings on TNF‐α secretion and pro‐inflammatory cytokine and chemokine signaling, we performed an NF‐κB reporter assay with Luc‐HCT‐116 colon cells in response to soy lecithin. NF‐κB activation starting at a dose of 0.39 mg/mL was observed (Figure 3B). Because of increased cell death, NF‐κB activation decreased at a dose of 1.56 mg/mL. In addition, we directly tested the effect of TNF pathway neutralization using anti‐TNF‐α in the barrier assay shown in Figure S1D Co‐treatment of soy lecithin 6.25 mg/mL with anti‐TNF‐α (2.5, 5, or 10 ng/mL) did not restore TER relative to soy lecithin 6.25 mg/mL alone. Thus, TNF neutralization under these tested conditions did not measurably mitigate the soy lecithin‐associated TER decline. Overall, the results indicate that soy lecithin stimulates innate immune responses, pro‐inflammatory cytokines, and chemokines in gut epithelial cells.

Since we observed transcriptomic differences in the genes related to the AhR response such as AhR and CYP1A1, we investigated the AhR response upon exposure to soy lecithin. The AhR response plays a major role in the response to several environmental toxic substances [48], and we found that treatment with the AhR reporter cell line with soy lecithin similarly resulted in a significant decrease in AhR activity, but only at high doses (Figure 3C), suggesting that soy lecithin may modulate AhR signaling. This reduction could be due to direct inhibition of AhR activation, interference with its dimerization or nuclear translocation, or suppression of endogenous AhR ligand production. It should be noted that cell death increases with high doses of soy lecithin, as observed in three different cell types (Figures 1A, 3B,C).

During our search to identify the mechanism of soy lecithin toxicity, we hypothesized that soy lecithin‐induced oxidative stress could play a role in NF‐κB activation. Soy lecithin caused oxidative stress at doses as low as 0.09 mg/mL (Figure 3D). Accordingly, we used N‐acetylcysteine (NAC), an antioxidant agent, to suppress the soy lecithin‐induced oxidative stress. NAC treatment together with soy lecithin decreased CCL20, CCL25, OPG, SIT1, DDX58, LIF, and TNFRSF9 levels compared to soy lecithin alone (Figure 3E). In addition, we evaluated the mitigation effect of NAC on the barrier assay shown in Figure S1D. Co‐treatment with NAC (1, 2, or 4 mM) during soy lecithin 6.25 mg/mL exposure did not improve TER relative to soy lecithin alone. Therefore, NAC did not alleviate the soy lecithin‐associated decline in TER, even if it was affecting the pro‐inflammatory levels in secreted proteins. Overall, these data elucidate the critical role of soy lecithin‐induced NF‐κB activation due to oxidative stress, followed by TNF release, which leads to cellular injury and pro‐inflammatory cytokine and chemokine production.

### 3.3 DATEM Induces Inflammation and Cell Death as Observed in Transcriptome and Proteome of Gut‐On‐a‐Chip

To assess the dose‐dependent effects of DATEM on the gut‐on‐a‐chip model, we performed transcriptomic analysis after 24 h, and exposure to 1 mg/mL of DATEM resulted in differential expression (*p* < 0.05) of 2384 genes (Figure S1A). A heatmap of the top 500 variable genes showed a dose‐dependent response (Figure S1B). Principal component analysis (PCA) reduced the dataset to two dimensions, with PC1 and PC2 accounting for 49.9% and 9.4% of the total variance, respectively. The PCA plot showed clear group clustering, indicating distinct effects on the transcriptome between them (Figure 4A). GSEA of 1 mg/mL DATEM exposure revealed significant upregulation of response to chemokine, TNF, ER stress, and NF‐kB signaling, and apoptosis, and downregulation of oxidative phosphorylation (Figure 4B,C).

**FIGURE 4:**
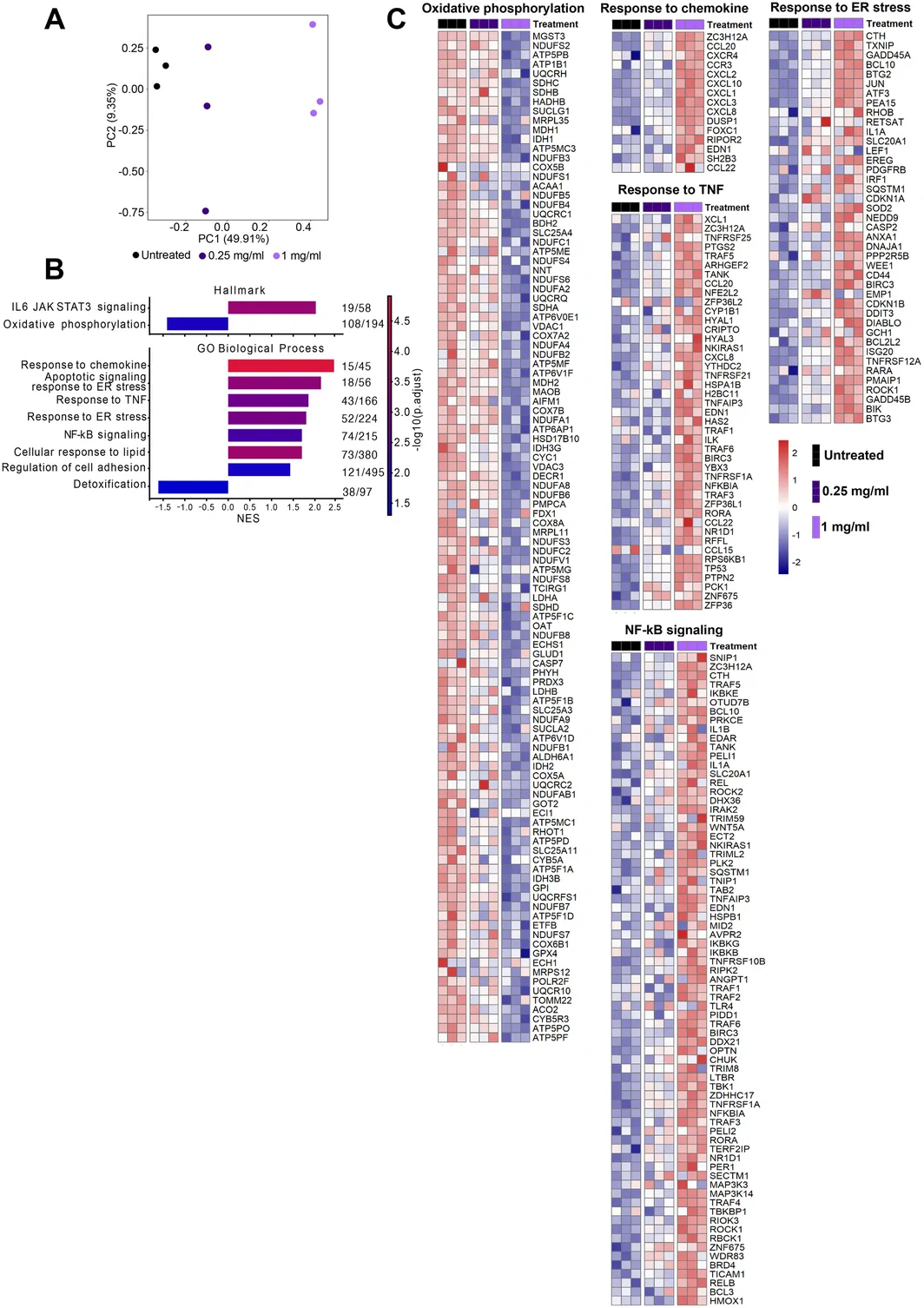
Transcriptome analysis of gut epithelial cells in response to DATEM in a gut‐on‐a‐chip model. (A) Principal component analysis (PCA) shows three distinct groups. The black dots represent control samples, while dark and light purple represent 0.25 mg/mL and 1 mg/mL, respectively. (B) GSEA of 1 mg/mL soy lecithin exposure. Hallmark and Gene Ontology Biological Process (GOBP) databases were used. IL‐6/JAK/STAT3 signaling (M5897), oxidative phosphorylation, response to chemokine (GO:1990868), Apoptotic signaling pathway (GO:0097190), Response to ER stress (GO:0034976), Response to TNF (GO:0034612), NF‐κB signaling (GO:0007249), Cellular response to lipid (GO:0071396), and regulation of cell adhesion (GO:0030155). (C) Sub‐heatmaps of the pathways and respective genes after 24 h of treatment with DATEM.

We performed targeted proteomics to assess proteome differences upon exposure to DATEM in the gut‐on‐a‐chip model with four different panels, namely, immune response, inflammation, metabolism, and organ damage (Figure 5A). In line with the transcriptomic results, targeted proteomic analysis showed increased secretion of TNF‐α and NF‐kB activation‐related mediators such as IL‐1 alpha, IL8, LIF, CCL20, CXCL1, AREG, JUN, CASP‐8, and AXIN1 (Figure 5A). In addition, we performed targeted proteomics in the human ASC‐derived colon organoid‐on‐a‐chip model and observed an increase in TNF‐α in response to DATEM (Figure S1C).

**FIGURE 5:**
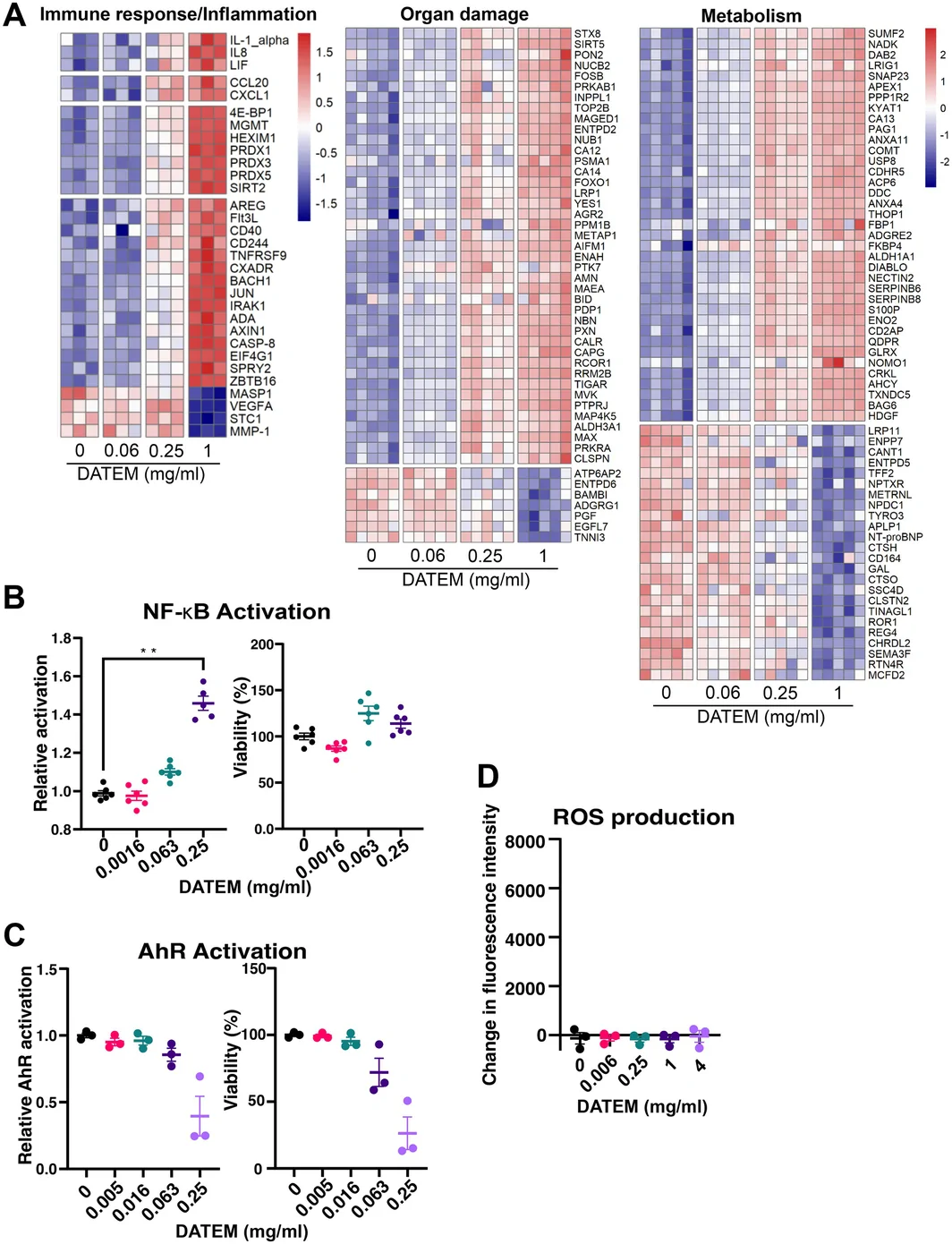
DATEM induces NF‐κB activation and inflammation. (A) Heatmap of targeted proteomic analysis of the gut‐on‐a‐chip model treated with DATEM. Immune response, inflammation, metabolism, and organ damage panels were used. (B) An NF‐κB activation assay was performed with the HCT‐116 reporter cell line. Cells were treated with DATEM for 24 h. Data are shown as relative NF‐κB stimulation. (C) An AhR activation assay was performed with HEK reporter cell line. Cells were treated with DATEM for 24 h. Data are shown as relative AhR stimulation. (D) Reactive oxygen species (ROS) release was performed with Caco‐2 cells. Cells were treated with DATEM for 6 h. Data are shown as change in fluorescence intensity. Data are presented as the mean ± SEM from three independent experiments conducted at different times. *p < 0.05, **p < 0.01, ***p < 0.001, ****p < 0.0001, Kruskal–Wallis test with Dunn's post hoc test.

To support whether the transcriptome and proteome data on TNF‐α secretion and pro‐inflammatory cytokine and chemokine signaling are related to NF‐κB activation, we performed an NF‐κB reporter assay with Luc‐HCT‐116 colon cells. DATEM induced NF‐κB activation at a dose of 0.25 mg/mL dose in the colon cells (Figure 5B). Overall, DATEM induces an innate immune response with pro‐inflammatory cytokine and chemokine release in gut epithelial cells.

To compare the findings with soy lecithin, we performed the same assays to determine AhR and oxidative stress responses. Treatment of the AhR reporter cell line with DATEM did not result in a statistically significant change in AhR activity (Figure 5C). These data suggest that DATEM does not directly impact AhR signaling under the tested conditions, or that any potential effects are subtle or context‐dependent. Likewise, there was no significant change in oxidative stress in response to DATEM (Figure 5D). These findings show the critical role of DATEM‐induced TNF‐α release, and NF‐κB activation, and pro‐inflammatory cytokine and chemokine production as its mechanisms of action.

In addition, to assess the effects of co‐exposure to DATEM and soy lecithin, we measured the expression of genes associated with xenobiotic signaling, inflammation, and epithelial function. Soy lecithin significantly increased CYP1A1 expression compared with control and DATEM‐treated conditions, consistent with activation of xenobiotic response pathways. Both emulsifiers upregulated inflammatory and stress‐associated genes, including TNFAIP3 and JUN. In contrast, DATEM preferentially elevated genes linked to epithelial remodeling and barrier function, including ITGA6, AREG, and ZO‐1. Notably, co‐exposure generally resulted in greater induction of these genes compared did the individual treatments, indicating additive or synergistic transcriptional effects.

### 3.4 Emulsifiers Cause an Inflammatory Response Throughout the Gastrointestinal Track

To assess the in vivo effects of both emulsifiers, mice received consumer‐relevant doses of emulsifiers once a week for 6 weeks (Figure S2A). We extracted total proteins from the esophagus, stomach, ileum, and colon and performed targeted proteomics. Figure 6A,B demonstrated the heatmap analysis of the significantly different proteins for soy lecithin and DATEM, respectively. Although all four tissues showed visible inflammation, DATEM appeared to be much more inflammatory compared to soy lecithin. A group of commonly regulated pro‐inflammatory proteins such as CCL17, TNF, CCL2, CXCL2, AhR, and PRDX5, were upregulated. In contrast, tissue response, stress response, cell proliferation and wound healing‐related proteins, such as TGF, GCG, FOXO1, PPP1R2, EDA2R, TNFRSF11B, SNAP29, and DCTN2 were downregulated. Interestingly, for both soy lecithin and DATEM, the number of significantly changing proteins increased toward the colon. The proteins that showed distinct expression between DATEM and soy lecithin were related to pathways such as signal transduction and cell chemotaxis, which were downregulated. One would expect to observe more pronounced effects in the esophagus since it is the first exposure point. However, we speculate that the effects of the emulsifiers accumulate toward the colon because of the slower intestinal transit and longer exposure time.

**FIGURE 6:**
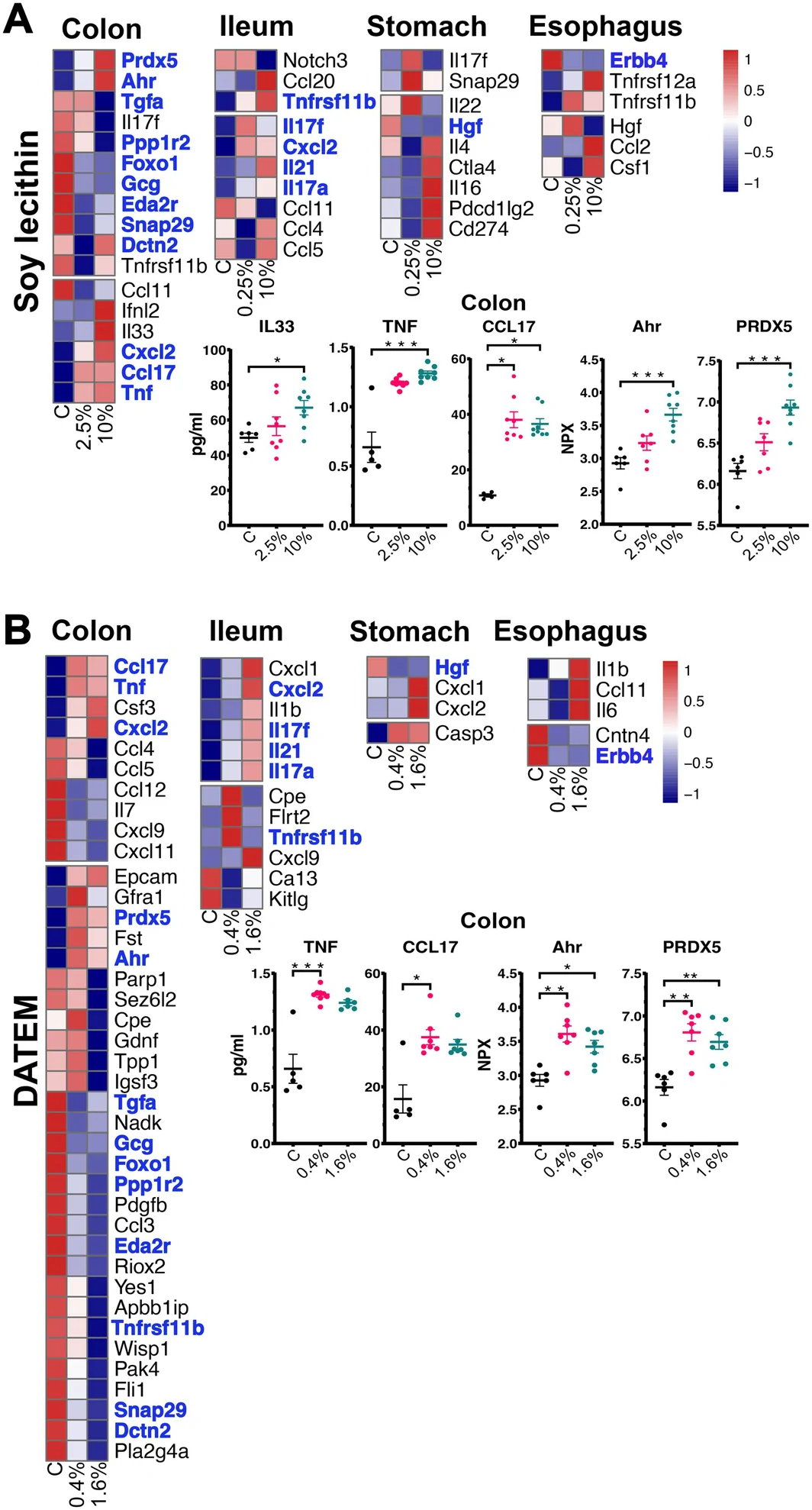
Emulsifiers induce inflammation throughout the gastrointestinal tract. Tissue samples from the colon, ileum, stomach, and esophagus exposed to soy lecithin (A) and DATEM (B) showed increased production of pro‐inflammatory mediators. Data are presented as the mean ± SEM from eight individual female BALB/c mice per condition. *p < 0.05, **p < 0.01, ***p < 0.001, ****p < 0.0001, Kruskal–Wallis test with Dunn's post hoc test.

Next, we performed RNA sequencing transcriptomics analysis of esophagus and colon tissue samples. GSEA of 10% soy lecithin exposure revealed significant upregulation of mast cell activation, IL‐6 production, NF‐κB signaling, response to TNF and apoptosis pathways and downregulation of keratinocyte differentiation and detoxification pathways in the esophagus (Figure S2B). However, the same dose of soy lecithin induced the upregulation of the unfolded protein response, inflammatory response, DNA repair pathways, and downregulation of intestinal lipid absorption and xenobiotic catabolic process pathways in the colon (Figure S2C). Additionally, GSEA of 1.6% DATEM exposure showed significant upregulation of TNF signaling, cell chemotaxis, lymphocyte anergy, pyroptosis and apoptosis pathways in the esophagus (Figure S2D). At the same dose of DATEM, we observed upregulation of TNF signaling, unfolded protein response, response to chemokines, IL‐4 production, and downregulation of glycolysis and oxidative phosphorylation in the colon (Figure S2E).

Overall, in vivo exposure to consumer‐relevant doses of soy lecithin and DATEM caused tissue‐specific molecular changes along the gastrointestinal tract, with proteomic alterations increasing toward the colon and transcriptomic analyses revealing distinct inflammatory and stress‐related pathway activations in both the esophagus and colon, including TNF signaling, apoptosis, and the unfolded protein response.

### 3.5 Increased Serum Levels of Pro‐Inflammatory Mediators in Emulsifier Exposure

To evaluate the systemic effects of both emulsifiers, we compared the serum levels of circulating pro‐inflammatory mediators between emulsifier‐fed mice and control mice. We observed that with soy lecithin exposure, the secretion of mediators such as IL‐10, IL‐3, IL‐6, CTLA‐4, IL1‐β, IFNL2, and TNF‐α increased (Figure 7A). Similarly, DATEM exposure showed increased secretion of IL10, IL17f, IL3, CCL4, IL6, IL1β, CTLA‐4, and TNF‐α (Figure 7B). Overall, systemic analysis of serum from emulsifier‐fed mice revealed that both soy lecithin and DATEM exposure led to increased serum levels of multiple inflammatory and immune‐related mediators, including IL‐6, IL‐1β, TNF‐α, and CTLA‐4, indicating a systemic pro‐inflammatory response.

**FIGURE 7:**
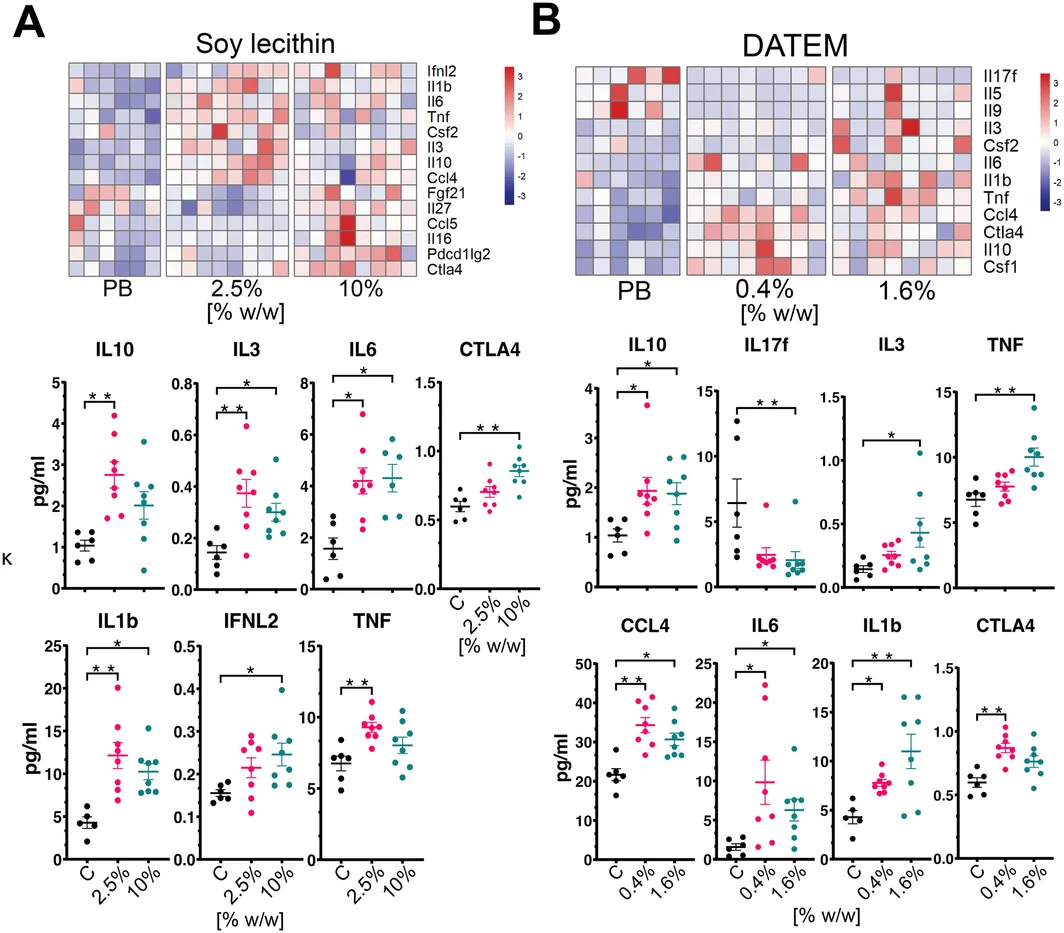
Emulsifiers induce systemic inflammation. Serum samples from mice exposed to soy lecithin (A) and DATEM (B) showed increased production of inflammatory mediators. Data are presented as the mean ± SEM from eight individual female BALB/c mice per condition. *p < 0.05, **p < 0.01, ***p < 0.001, ****p < 0.0001, Kruskal–Wallis test with Dunn's post hoc test.

### 3.6 DATEM and Soy Lecithin Both Induced IgG and IgE in PBMCs

To investigate the effects of both emulsifiers on total immunoglobulins in vivo, we checked total IgG and total IgE levels in serum samples from emulsifier‐fed mice after 6 weeks. We observed increased levels of both total IgG and total IgE in response to soy lecithin, whereas DATEM exerted an increasing trend in total IgG levels (Figure 8A).

**FIGURE 8:**
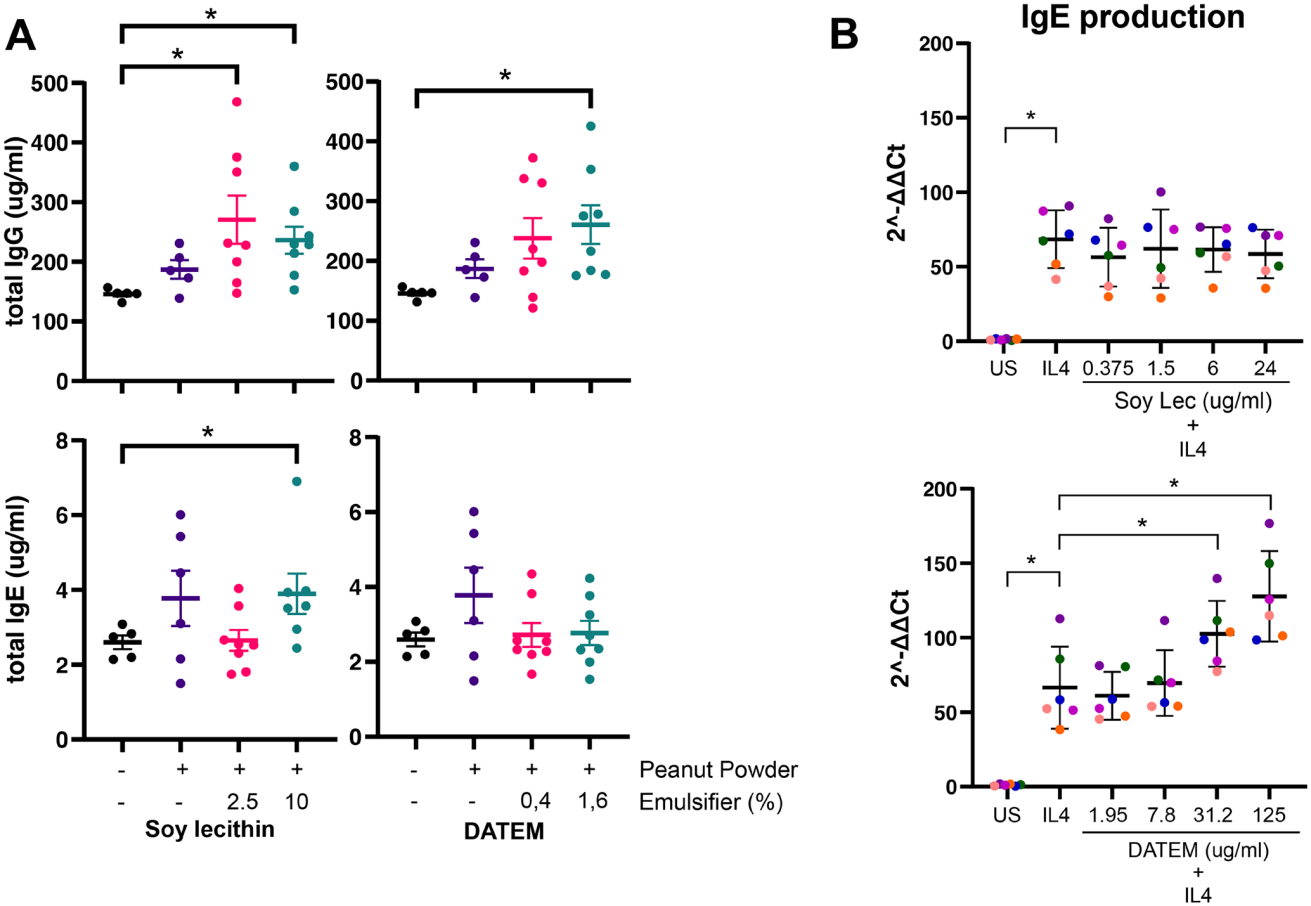
DATEM induces the production of IgE in PBMCs. (A) Total IgG and total IgE levels in mice fed soy lecithin and DATEM. Data are presented as the mean ± SEM from eight individual female BALB/c mice per condition. (B) PBMCs were treated with IL‐4 with or without emulsifiers for 5 days, and IgE induction was assessed by RT‐qPCR. Data are presented as the mean ± SEM from four individual healthy donors. *p < 0.05, **p < 0.01, ***p < 0.001, ****p < 0.0001, Kruskal–Wallis test with Dunn's post hoc test.

Next, we treated human PBMCs with both emulsifiers to check their IgE‐inducing capacity. As expected, IL‐4 stimulated IgE mRNA production in PBMCs. The addition of DATEM to IL‐4 induced a dose response in IgE mRNA expression, while soy lecithin showed no significant effect (Figure 8B).

Overall, these findings showed that the consumption of soy lecithin may lead to the induction of IgE in vivo while DATEM may potentially increase IgE production in vitro, *indicating the allergy development* capability of both emulsifiers.

## 4 Discussion

In the present study, we demonstrate that the widely used food emulsifiers, soy lecithin and DATEM, induce cellular cytotoxicity, local and systemic inflammation, and impair gastrointestinal epithelial barrier integrity in both in vitro and in vivo models, at concentrations reflective of daily‐life exposure. Given the essential role of the intestinal epithelial barrier in immune surveillance and microbial containment, these findings are of significant concern [49]. The observed periepithelial inflammation and barrier dysfunction imply increased intestinal permeability, namely, leaky gut, a phenomenon increasingly associated with allergic, autoimmune, and metabolic diseases [50]. These results suggest that commercial usage of emulsifiers that are currently considered “safe” can compromise epithelial barrier integrity and potentially facilitate systemic inflammation and immune activation.

The present study focused on human in vivo–relevant 3D models and the effects of the two emulsifiers were investigated in human gut‐on‐a‐chip, adult stem cell‐derived colon organoid‐on‐a‐chip, as well as by direct feeding of mice. The findings were consistent in all the studied models. Soy lecithin significantly upregulated genes involved in TNF signaling, unfolded protein response, apoptosis, and inflammation, consistent with epithelial stress and immune activation [51]. Oxidative stress emerged as a key mechanism with soy lecithin inducing ROS production at sub‐cytotoxic concentrations. Co‐treatment with the antioxidant, NAC, reduced pro‐inflammatory cytokine secretion, confirming ROS involvement in NF‐κB activation [52]. Notably, NAC did not overcome the barrier disruption capacity of soy lecithin, suggesting that soy lecithin‐mediated barrier disruption is mechanistically distinct from its ROS‐dependent inflammatory effects. In addition, ER stress and the unfolded protein response potentiated inflammatory signaling and promoted apoptosis under sustained stress conditions. Negative regulation of the AhR response further suggested the loss of a key epithelial anti‐inflammatory regulatory pathway, amplifying immune activation. Overall, data from this study identified multiple cellular toxicity and pro‐inflammatory mechanisms induced by soy lecithin.

DATEM similarly induced strong transcriptional and proteomic pro‐inflammatory changes, including elevated TNF‐α, IL‐8, and CASP‐8 expression, which are indicative of canonical TNF receptor‐mediated signaling and engagement of extrinsic apoptotic pathways, although it did not elicit oxidative stress or influence AhR activity. These observations indicate that while the two emulsifiers may act through distinct upstream mechanisms, both converge on NF‐κB signaling and pro‐inflammatory cytokine production, a hallmark of innate immune activation [53, 54]. Since dietary emulsifiers are commonly consumed in combination, we investigated the effects of co‐exposure to soy lecithin and DATEM on epithelial gene expression. The results amplify epithelial transcriptional responses, suggesting that combined emulsifier intake may exacerbate inflammatory and remodeling pathways beyond those of individual effects.

Systemic effects were evident in emulsifier‐treated mice, with serum proteomics showing increased levels of inflammatory mediators such as IL‐6, TNF, IL‐1β, and IL‐10. Specifically, increased IL‐33 levels together with multiple cytokines and chemokines in tissues exert the evidence of epithelitis [15]. These data demonstrate that “circulating inflammation increases with exposure to both emulsifiers,” a newly developing concept leading to systemic inflammation because of compromised epithelial barrier function, a key feature of many non‐communicable diseases [15, 55]. Interestingly, the degree of proteomic alteration increased throughout the gastrointestinal passage from the esophagus to the colon, suggesting that cumulative exposure takes place or that there is heightened sensitivity of the distal gut to emulsifier‐induced damage. Although the esophagus is the first exposed organ, toward the colon the speed of the passage slows down and exposure time increases [56, 57].

It remains to be elucidated whether microbiome changes play a role in these inflammatory effects. While the harmful effects of synthetic emulsifiers on gut microbiota and barrier function are well‐documented [28, 58, 59], fewer studies have assessed natural or industry‐preferred emulsifiers, such as soy lecithin and DATEM and their effects on the microbiome and epithelial barrier. DATEM appeared to be much more inflammatory compared to soy lecithin and displayed strong immunostimulatory properties. It increased total IgG levels in vivo and enhanced IL‐4‐induced IgE production in human PBMCs, suggesting a role in promoting type 2 immune responses and allergic sensitization [60]. This area needs further research as the current findings suggest a strong role for the increased prevalence of FA particularly. In addition, these findings demonstrate the adjuvant effect of the two emulsifiers that may activate B cells and play a role in the induction of IgG and IgE. Moreover, their capacity to amplify the total IgG response for both emulsifiers and IgE for DATEM raises concerns about their role in sensitization to food allergens. This is especially relevant given current regulatory policies such as the “quantum satis” principle that allow unrestricted emulsifier usage within good manufacturing practice limits [47].

Overall these data align with the epithelial barrier theory, which posits that environmental agents such as detergents, emulsifiers, and surfactants can disrupt mucosal barriers, increasing antigen exposure and facilitating allergy development [14]. Findings, such as inflammation, adjuvanticity, induction of IgE and IgG, warrant attention considering the rising incidence of allergic and autoimmune diseases potentially linked to environmental and dietary exposures [58]. Both soy lecithin and DATEM have long been classified as safe based on traditional toxicological assessments, which primarily evaluate acute toxicity and carcinogenicity. However, our data suggest that these models may overlook chronic, low‐grade inflammatory responses and mucosal barrier dysfunction. A recent report emphasizes the need for updated safety frameworks that incorporate effects on gut barrier function, microbiota, and immune signaling [61].

The present study has some limitations. We did not determine the exact local concentration of emulsifiers reaching the gut. Consequently, we utilized concentrations corresponding to reported use levels found in foods and beverages. Another limitation of this study is that a chronic in vivo exposure model is missing. It is expected that the multiple mechanisms of cellular distress caused by the daily doses in quite a short time (one time per week for 6 weeks) could reflect chronic exposure even at lower doses. It has to be emphasized here that these exposures often happen together with other toxic substances, such as air pollutants, detergents, and rinse aids that have also been proven to show cellular toxicity. Another key point to be considered in future studies is the genetic tendency of the individuals and the effects of the emulsifiers on gut microbiota to disentangle epithelial‐intrinsic versus microbiome‐dependent contributions.

The widespread use of emulsifiers in ultra‐processed foods and the cumulative nature of exposure may pose serious public health implications. The combination of epithelial damage, local inflammation, and systemic cytokine elevation suggests a plausible pathway linking emulsifier consumption to chronic diseases such as inflammatory bowel disease (IBD), obesity, and metabolic syndrome [62, 63]. These findings underscore the need for reevaluating food additive safety using models that capture chronic exposure and biologically relevant endpoints. Regulatory agencies should consider revising risk assessment frameworks to include effects on gut permeability, immune activation, and microbiome alterations. At the public health level, consumer education and reformulation of processed foods with safer alternatives could mitigate potential risks. Future research should focus on long‐term health outcomes, microbiome‐emulsifier interactions, and the identification of safer food formulation strategies.

## Funding

This work was supported by the Swiss National Science Foundation and the EU project, SynAir‐G (project no. 101057271).

## Conflicts of Interest

M.A. has received research grants from the Swiss National Science Foundation, Bern; research grant from Stanford University; Leading House for the Latin American Region, Seed Money Grant. She is the Scientific Advisory Board member of Stanford University Sean Parker Asthma Allergy Center, CA; Advisory Board member of LEO Foundation Skin Immunology Research Center, Copenhagen; and Scientific Co‐Chair of World Allergy Congress (WAC) Istanbul, 2022, Scientific Programme Committee Chair, EAACI. K.N. currently reports grants from National Institute of Allergy and Infectious Diseases (NIAID), National Heart, Lung, and Blood Institute (NHLBI), National Institute of Environmental Health Sciences (NIEHS); Stock options from IgGenix, Seed Health, ClostraBio, Cour, Alladapt; Consultant for Excellergy, Red Tree Ventures, Regeneron, and IgGenix; Co‐founder of Alladapt, Latitude, and IgGenix; National Scientific Committee member at Immune Tolerance Network (ITN), and National Institutes of Health (NIH) clinical research centers; patents include, “Mixed allergen composition and methods for using the same,” “Granulocyte‐based methods for detecting and monitoring immune system disorders,” and “Methods and Assays for Detecting and Quantifying Pure Subpopulations of White Blood Cells in Immune System Disorders.” O.P. has received research grants from MINECO, Ministerio de Ciencia e Innovación, CAM, Inmunotek S.L., Novartis, and AstraZeneca and fees for giving scientific lectures or participation in Advisory Boards from AstraZeneca, Pfizer, GlaxoSmithKline, Inmunotek S.L, Novartis, Sanofi‐Genzyme and Regeneron. C.A.A. has received research grants from the Swiss National Science Foundation, European Union (EU CURE, EU Syn‐Air‐G), Novartis Research Institutes (Basel, Switzerland), Stanford University (Redwood City, Calif), Seed Health (Boston, USA) and SciBase (Stockholm, Sweden); is the Co‐Chair for EAACI Guidelines on Environmental Science in Allergic diseases and Asthma; is on the Advisory Boards of Sanofi/Regeneron (Bern, Switzerland, New York, USA), Stanford University Sean Parker Asthma Allergy Center (CA, USA), Novartis (Basel, Switzerland), Glaxo Smith Kline (Zurich, Switzerland), Bristol‐Myers Squibb (New York, USA), Seed Health (Boston, USA), and SciBase (Stockholm, Sweden); and is the Editor‐in‐Chief of Allergy. I.O. is chair of the EAACI Epithelial Cell Biology Working Group. N.S. has received grants from CSC scholarship program of China (No. 202008210164). R.D. is a non‐executive Board Director at Seed Health Inc. The remaining authors D.Y., Y.H., Y.P., J.H., M.A.B., S.A., J.F.L., O.A., B.Z., A.M., C.Z., K.M., M.L., B.R., L.C., V.E. and H.M. declares no conflicts of interest.

## Supporting information


**Figure S1:** (A) Top 500 variable genes after treatment with soy lecithin and DATEM. (B) Volcano plots showing upregulated and downregulated genes after treatment of soy lecithin and DATEM. (C) Heatmaps showing targeted proteomics analysis of soy lecithin and DATEM‐treated colon organoid‐on‐a‐chip with immune response and inflammation panels. (D). Epithelial barrier mitigation experiment with anti‐TNF‐α antibody and NAC, NAC: n‐acetylcysteine, TER: transepithelial electrical resistance, E. Relative gene expression levels of CYP1A1, TNFAIP3, JUN, ITGA6, AREG, and ZO1 following exposure to soy lecithin (Soy Lec), DATEM, or their combination. Expression values are shown as arbitrary units normalized to EF1α. Bars represent mean ± SEM with individual data points overlaid. *p* < 0.05 (*) and *p* < 0.01 (**).
**Figure S2:** (A) Experimental plan of in vivo emulsifier treatment, GSEA of esophagus (B) and colon (C) tissues prior to soy lecithin treatment in vivo, GSEA of esophagus (D) and colon (E) tissues prior to DATEM treatment in vivo. Interferon gamma response (M5913), IL6 JAK–STAT signaling (M5897), IL2–STAT5 signaling (M5947), Epithelial–mesenchymal transition (M5930), Unfolded protein response (M5922), Inflammatory response (M5932), Cholesterol homeostasis (M5892), TNF‐α signaling via Nf‐kB (M5890), Glycolysis (M5937), Oxidative Phosphorylation (M5936), T cell activation (GO:0042110), Complement Activation (GO:0006956), Phagocytosis (GO:0006909), Positive regulation of cell adhesion (GO:0045785), Cell chemotaxis (GO:0060326), Fc receptor signaling pathway (GO:0038093), Mast cell activation (GO:0045576), Cytokine mediated signaling pathway (GO:0019221), Interleukin‐16 production (GO:0032619), Nf‐kB signaling (GO:0007249), Positive regulation of epithelial cell apoptotic process (GO:1904037), Response to TNF (GO:0034612), Hypersensitivity (GO:0002524), Response to wounding (GO:0009611), Keratinocyte differentiation (GO:0030216), Detoxification (GO:0098754), Lipid oxidation (GO:0034440), Fatty acid catabolic process (GO:0009062), Protein folding (GO:0006457), Regulation of cell surface receptor signaling pathway (GO:0007166), Activation of immune response (GO:0002253), Adaptive immune response (GO:0002250), DNA repair (GO:0006281), Triglyceride metabolic process (GO:0006641), Fatty acid metabolic process (GO:0006631), Intestinal lipid absorption (GO:0098856), Xenobiotic catabolic process (GO:0042178), Interleukin 10 production (GO:0032613), Lymphocyte anergy (GO:0002249), Eosinophil migration (GO:0072679), Pyroptosis (GO:0070269), Regulation of epithelial cell apoptotic process (GO:1904035), ERK1 and ERK2 cascade (GO:0070371), IkB kinase/NF‐kB signaling (GO:0033256), Response to chemokine (GO:1990868), Regulation of cell‐cell adhesion (GO:0022407), Interleukin 4 production (GO:0032633), Positive regulation of double strand break repair (GO:2000781), Phospholipid biosynthetic process (GO:0008654), Mitochondrial respiratory chain complex assembly (GO:0033108). Eight individual female BALB/c mice were used per condition.
**Table S1:** Usage history of soy lecithin and DATEM in peanut butter formulations.
**Table S2:** Peanut butter preparation.
**Table S3:** Doses are estimated daily exposure to emulsifiers. The doses were selected according to the evaluations of the European Food Safety Authority (EFSA).
**Table S4:** Reported usage of lecithin (E322) and DATEM (E472f) across food categories.

## Data Availability

The data that support the findings of this study are available from the corresponding author upon reasonable request.
