# Supplementary material for: Common Food Emulsifiers Impair Epithelial Barrier Integrity and Trigger Pro‐Inflammatory Responses and IgE Production
Source: Allergy. 2026 Aug 8;81(10):3641–58. doi: 10.1111/all.70473 (PMC13646865; doi:10.1111/all.70473)
Supplement: Supplementary file 1 [file ALL-81-3641-s001.docx]

**Supplementary Methods**

***Coculture of DATEM and soy lecithin***

Caco-2 cells were cultivated in 96-well plates at a density of 25,000 cells/well and incubated overnight at 37°C in a humidified incubator with 5% CO_2_. 1 mg/ml of DATEM and 6.25 mg/ml of soy lecithin were applied alone and together to cultures for 24 hours. Total RNAs were extracted and purified with the RNeasy Plus Micro Kit (Qiagen, Hilden, Germany) according to the manufacturer’s protocol. NanoDrop 2000 spectrophotometer (Thermo Fisher Scientific) were used to measure RNA concentration. cDNA synthesis was done with RevertAid First Strand cDNA Synthesis Kit (Thermo Fisher Scientific) according to the manufacturer’s protocol. Real-time PCR reaction was performed by QuantStudio™ 7 Flex Real-Time PCR System (Thermo Fisher). The sequences of primers are as follows: CYP1A1 forward: ACA TGC TGA CCC TGG GAA AG, CYP1A1 reverse: GGT GTG GAG CCA ATT CGG AT, TNFAIP3 forward: TTG TCC TCA GTT TCG GGA GAT, TNFAIP3 reverse: ACT TCT CGA CAC CAG TTG AGT T, JUN forward: AAC AGG TGG CAC AGC TTA AAC, JUN reverse: CAA CTG CTG CGT TAG CAT GAG, ITGA6 forward: TTT GAA GAT GGG CCT TAT GAA, ITGA6 reverse: CCC TGA GTC CAA AGA AAA ACC, AREG forward: ACA CCT ACT CTG GGA AGC GT, AREG reverse: GCC AGG TAT TTG TGG TTC GT, ZO1 forward: CGG GAC TGT TGG TAT TGG CTA GA, ZO1 reverse: GCT AGG CCA GGG CCA TAG TAA AG and EF1α forward: CTG AAC CAT CCA GGC CAA AT, EF1α reverse: GCC GTG TGG CAA TCC AAT.

For mitigation experiments, cells were pretreated with 1, 2, and 4 mM n‐acetylcysteine (NAC) (Sigma‐Aldrich) and 2.5, 5, and 10 ng/ml anti‐TNF‐α antibody (Sigma‐Aldrich) for 1 h and emulsifiers were added for the rest of 4 days together with mitigating agents.

**Rationale for Emulsifier Concentrations Used in the Mouse Model**

In our mouse model, each animal received 3 g of peanut butter containing 0.4% DATEM and 2.5% soy lecithin per administration, once per week for six weeks. Assuming a consumption of approximately 3 g peanut butter per mouse and an average body weight of 25 g, this corresponds to approximately 12 mg DATEM (~480 mg/kg body weight) and 75 mg soy lecithin (~3000 mg/kg body weight). Using standard body surface area conversion for interspecies dose scaling (FDA guidance, Reagan-Shaw et al., 2008), these exposures correspond to human equivalent doses of approximately 39 mg/kg (~2.7 g/day) for DATEM and ~243 mg/kg (~17 g/day) for soy lecithin in a 70-kg adult. While the lecithin dose exceeds exposure from a single food product, it reflects the upper range of cumulative dietary exposure, as lecithin is widely used as an emulsifier in chocolate, baked goods, processed snacks, margarines, dressings, and other manufactured foods (EFSA, Younes et al., 2020). In addition, rodent dietary studies investigating food additives commonly use concentrations between 0.1–5% of the diet to model chronic exposure and to detect mechanistic effects, partly because rodents have higher metabolic rates and food intake per body weight than humans. Therefore, the concentrations used in the present study fall within the range typically employed in experimental dietary exposure models investigating the biological effects of food emulsifiers.

***Data and statistical analyses***

All statistical analyses and associated figures were generated using GraphPad Prism software (version 9.0) or R package. Differences among groups were analyzed using the Kruskal-Wallis test followed by Dunn’s post hoc and P value < 0.05 was considered statistically significant.

The raw sequencing reads in “.fastq” format were initially processed to obtain analysis-ready clean reads using Trimmomatic v0.39 (Bolger et al., 2014) and following criteria; (i) removal of Illumina adapter sequences, (ii) filtering of low-quality reads (< Q20, Phred score), (iii) removal of reads with a base length below 30 bp. The processed reads were evaluated for sequence quality, GC content, base N content, and adapter contamination using FastQC v0.12 (Andrews, 2010). The quality control reports were subsequently compiled with MultiQC v1.23 (Ewels et al., 2016). The pre-processed reads were mapped to the human reference genome (GRCh38.p14) and the mouse reference genome (GRCm39, Ensembl release M30) using HISAT2 v2.2.1 (Kim et al., 2019) with default parameters. The resulting alignments in “.sam” format were converted to the more compact “.bam” format and then sorted using Samtools v1.20 (Li et al., 2009). To evaluate alignment quality, metrics such as exonic rate, intronic rate, and overall alignment rate were computed using RNA-SeQC v2.4.2 (Graubert et al., 2021). Gene expression levels were quantified using featureCounts v2.0.3 (Liao et al., 2013), resulting in an expression matrix for downstream analysis. Group-wise concordance in gene expression was assessed via principal component analysis (PCA), visualized with ggplot2 v3.5.1 (Wickham, 2016). Differentially expressed genes between conditions were identified using DESeq2 v1.44.0 (Love et al., 2014). Genes were considered significantly differentially expressed if they met the thresholds of log2 fold change (log2FC > 1 for upregulation and log2FC < -1 for downregulation) and a Benjamini-Hochberg adjusted p-value of <0.05. Gene expression differences across groups were visualized with Pheatmap v1.0.12 (Kolde, 2018). Gene Set Enrichment Analysis (GSEA) was performed using the clusterProfiler R package (Yu et al., 2012) to identify significantly enriched gene sets in the Molecular Signatures Database (MSigDB). Genes were ranked by the Wald test statistic from DESeq2. Enrichment analysis was performed with a Benjamini-Hochberg adjusted p-value threshold of <0.05, including hallmark pathways, curated gene sets, and ontologies within MSigDB. Visualizations of enriched pathways were created using ggplot2 v3.5.1 (Wickham, 2016).

Proximity extension assay data were analysed using R (version 4.2.0). Differential expression analysis of proteins was achieved using the OlinkAnalyze package (version 3.8.2). Benjamini–Hochberg's false discovery rate corrected q-values were calculated to correct for multiple testing in all parts of the differential expression analysis. Proteins with an adjusted p value <.05 were considered significantly differential biomarkers.

**Supplementary Figure 1. A,** Top 500 variable genes after treatment of soy lecithin and DATEM, **B,** Volcano plots showing upregulated and downregulated genes after treatment of soy lecithin and DATEM, **C,** Heatmaps showing targeted proteomics analysis of soy lecithin and DATEM-treated colon organoid-on-a-chips with immune response and inflammation panels. **D.** Epithelial barrier mitigation experiment with anti‐TNF‐α antibody and NAC, NAC: n-acetylcysteine, TER: transepithelial electrical resistance, **E.** Relative gene expression levels of CYP1A1, TNFAIP3, JUN, ITGA6, AREG, and ZO1 following exposure to soy lecithin (Soy Lec), DATEM, or their combination. Expression values are shown as arbitrary units normalized to EF1α. Bars represent mean ± SEM with individual data points overlaid. p < 0.05 (*) and p < 0.01 (**).

**Supplementary Figure 2. A,** Experimental plan of in vivo emulsifier treatment, GSEA of esophagus **(B)** and colon **(C)** tissues prior to soy lecithin treatment in vivo**,** GSEA of esophagus (D) and colon (E) tissues prior to DATEM treatment in vivo. Interferon gamma response (M5913), IL6 JAK-STAT signaling (M5897), IL2-STAT5 signaling (M5947), Epithelial mesenchymal transition (M5930), Unfolded protein response (M5922), Inflammatory response (M5932), Cholesterol homeostasis (M5892), TNFa signaling via NfkB (M5890), Glycolysis (M5937), Oxidative Phosphorylation (M5936), T cell activation (GO:0042110), Complement Activation (GO:0006956), Phagocytosis (GO:0006909), Positive regulation of cell adhesion (GO:0045785), Cell chemotaxis (GO:0060326), Fc receptor signaling pathway (GO:0038093), Mast cell activation (GO:0045576), Cytokine mediated signaling pathway (GO:0019221), Interleukin-16 production (GO:0032619), Nf-kB signaling (GO:0007249), Positive regulation of epithelial cell apoptotic process (GO:1904037), Response to TNF (GO:0034612), Hypersensitivity (GO:0002524), Response to wounding (GO:0009611), Keratinocyte differentiation (GO:0030216), Detoxification (GO:0098754), Lipid oxidation (GO:0034440), Fatty acid catabolic process (GO:0009062), Protein Folding (GO:0006457), Regulation of cell surface receptor signaling pathway (GO:0007166), Activation of immune response (GO:0002253), Adaptive immune response (GO:0002250), DNA repair (GO:0006281), Triglyceride metabolic process (GO:0006641), Fatty acid metabolic process (GO:0006631), Intestinal lipid absorption (GO:0098856), Xenobiotic catabolic process (GO:0042178), Interleukin 10 production (GO:0032613), Lymphocyte anergy (GO:0002249), Eosinophil migration (GO:0072679), Pyroptosis (GO:0070269), Regulation of epithelial cell apoptotic process (GO:1904035), ERK1 and ERK2 cascade (GO:0070371), I kappab kinase Nf kappa signaling (GO:0033256), Response to chemokine (GO:1990868), Regulation of cell cell adhesion (GO:0022407), Interleukin 4 production (GO:0032633), Positive regulation of double strand break repair (GO:2000781), Phospholipid biosynthetic process (GO:0008654), Mitochondrial respiratory chain complex assembly (GO:0033108), 8 individual female balb-c mice were used per condition

**Supplementary table 1.** Usage history of soy lecithin and DATEM in peanut butter formulations.

|  | **Soy lecithin** | **DATEM** |
| --- | --- | --- |
| Before 1960s | Rare; early patents show lecithin as an idea, but mainstream PB used hydrogenated oils. | Not used in PB; DATEM invented mid-century for bread, not yet in spreads. |
| Between 1960-2000 | Widespread globally. Many commercial PBs contain small lecithin additions for stability. Noted as a typical ingredient. Usage in all sectors (retail, foodservice, etc.) grows. | *Initial adoption begins at 1990s.* A few lower-fat or trans-fat-free peanut butter spreads start to include DATEM as part of new stabilizer systems. Adoption still niche – e.g. perhaps in institutional formulations or novel products. |
| After 2000s | Universally accepted as a safe, “label-friendly” emulsifier. Regional note: U.S. major brands often still omit lecithin (relying on mono/diglycerides), but European brands commonly list soy lecithin (E322) as stabilizer. Also used in foodservice PB and industrial peanut pastes to ensure consistency. | Moderate use in specialized contexts. DATEM is utilized in some modern “clean label” or improved-texture peanut butter products but remains far from universal. It is widespread only in a broader sense (being found across various food industries); in peanut butter jars it’s relatively uncommon. |

PB: peanut butter

**Supplementary table 2.** Peanut butter preparation

|  | **PB powder (g)** | **Water (ml)** | **Emulsifier (g)** | **Total volume (ml)** |
| --- | --- | --- | --- | --- |
| Soy Lecithin 10% | 128 | 182 | 30 | 340 |
| Soy Lecithin 2.5% | 137 | 195 | 8 | 340 |
| DATEM 1.6% | 138 | 197 | 5 | 340 |
| DATEM 0.4% | 139 | 199.6 | 1.4 | 340 |
| Only PB | 140 | 200 | - | 340 |

PB: peanut butter

**Supplementary table 3.** Doses and estimated exposure amount of emulsifiers per day. The doses are selected according to the evaluations of the European Food Safety Authority (EFSA).

| **Compounds** | **Percentage of emulsifiers in PB powder** | **Exposure week** | **# of mice** |
| --- | --- | --- | --- |
| DATEM | PB + 0.4% w/w | 6 | 8 |
| DATEM | PB + 1.6% w/w | 6 | 8 |
| Soy Lecithin | 2.5% w/w | 6 | 8 |
| Soy Lecithin | 10% w/w | 6 | 8 |
| Placebo control 1 | only PB | 6 | 6 |
| Placebo control 2 | Normal food | 6 | 6 |

PB: peanut butter

**Supplementary table 4.** Reported usage of lecithin (E322) and DATEM (E472f) across food categories.

|  | **Lecithin (E322)** | | | | **DATEM (E472f)** | | | |
| --- | --- | --- | --- | --- | --- | --- | --- | --- |
| **Food category name** | **Maximum permitted levels** | **Reported use levels (mg/kg or mL/kg as appropriate)** | | **Percentage of products that have E322** | **Maximum permitted levels** | **Reported use levels (mg/kg or mL/kg as appropriate)** | | **Percentage of products that have E472f** |
|  |  | **Mean** | **Max** |  |  | **Mean** | **Max** |  |
| Nut butters and nut spreads | QS | 1,000 | 10,000 | 20.0 | QS | - | - | 0.65 |
| Cocoa and chocolate products ascovered by Directive 2000/36/EC | QS | 6,894 | 12,283 | 85.0 | QS | 3000 | 5000 | - |
| Other confectionery including breath freshening microsweets | QS | 862 | 7,000 |  | QS | 5000 | 5000 | 5.82 |
| Chewing gum | QS | 13,600 | 50,000 | 71.2 | QS | 30000 | 30000 | 0.09 |
| Bread and rolls | QS | 887 | 5,000 | 6.1 | QS | 3000 | 4000 | 14.8 |
| Processed Meat | QS | 165 | 318 | - | QS | 5000 | 25000 | 0.44 |
| Soups and broths | QS | 336 | 2,839 | - | QS | 1000 | 2000 | 1.2 |
| Sauces | QS | 1,459 | 10,688 | 0.5 | QS | 2000 | 5000 | 0.86 |
| Food supplements | QS | 4,002 | 18,387 | - | QS | 56200 | 56200 | 0.03 |

Maximum permitted levels are expressed as “quantum satis” (QS). Reported mean and maximum use levels (mg/kg or mL/kg) illustrate typical applications in different product categories.

**References**

Andrews, S. (2010). FastQC: A Quality Control Tool for High Throughput Sequence Data [Online].

Blighe K, Rana S, Lewis M (2024). EnhancedVolcano: Publication-ready volcano plots with enhanced colouring and labeling. R package version 1.22.0, <https://github.com/kevinblighe/EnhancedVolcano>.

Bolger, A. M., Lohse, M., & Usadel, B. (2014). Trimmomatic: a flexible trimmer for Illumina sequence data. Bioinformatics, 30(15), 2114–2120. <https://doi.org/10.1093/bioinformatics/btu170>

Ewels, P., Magnusson, M., Lundin, S., & Käller, M. (2016). MultiQC: summarize analysis results for multiple tools and samples in a single report. Bioinformatics, 32(19), 3047–3048. <https://doi.org/10.1093/bioinformatics/btw354>

Graubert, A., Aguet, F., Ravi, A., Ardlie, K. G., & Getz, G. (2021). RNA-SeQC 2: efficient RNA-seq quality control and quantification for large cohorts. Bioinformatics, 37(18), 3048–3050. <https://doi.org/10.1093/bioinformatics/btab135>

Kim, D., Paggi, J. M., Park, C., Bennett, C., & Salzberg, S. L. (2019). Graph-based genome alignment and genotyping with HISAT2 and HISAT-genotype. Nature Biotechnology, 37(8), 907–915. <https://doi.org/10.1038/s41587-019-0201-4>

Kolde R (2018). pheatmap: Pretty Heatmaps. R package version 1.0.12, <https://github.com/raivokolde/pheatmap>.

Liao, Y., Smyth, G. K., & Shi, W. (2013). featureCounts: an efficient general purpose program for assigning sequence reads to genomic features. Bioinformatics, 30(7), 923–930. <https://doi.org/10.1093/bioinformatics/btt656>

Wickham H (2016). ggplot2: Elegant Graphics for Data Analysis. Springer-Verlag New York. ISBN 978-3-319-24277-4, <https://ggplot2.tidyverse.org>.

Liu, Y., & Li, G. (2023). Empowering biologists to decode omics data: the Genekitr R package and web server. BMC Bioinformatics, 24(1). <https://doi.org/10.1186/s12859-023-05342-9>

Li, H., Handsaker, B., Wysoker, A., Fennell, T., Ruan, J., Homer, N., Marth, G., Abecasis, G., & Durbin, R. (2009). The Sequence Alignment/Map format and SAMtools. Bioinformatics, 25(16), 2078–2079. <https://doi.org/10.1093/bioinformatics/btp352>

Love, M. I., Huber, W., & Anders, S. (2014). Moderated estimation of fold change and dispersion for RNA-seq data with DESeq2. Genome biology, 15, 1-21.

Marini, F., & Binder, H. (2019). pcaExplorer: an R/Bioconductor package for interacting with RNA-seq principal components. BMC bioinformatics, 20, 1-8.

Reagan-Shaw, S., Nihal, M., and Ahmad, N. (2008). Dose translation from animal to human studies revisited. Faseb j 22, 659-661. 10.1096/fj.07-9574LSF.

Younes, M., Aquilina, G., Castle, L., Engel, K.-H., Fowler, P., Frutos Fernandez, M.J., Fürst, P., Gürtler, R., et al. (2020). Opinion on the re-evaluation of lecithins (E 322) as a food additive in foods for infants below 16 weeks of age and follow-up of its re-evaluation as food additive for uses in foods for all population groups. EFSA Journal 18, e06266. https://doi.org/10.2903/j.efsa.2020.6266.
